# Magnetic ligand fishing using immobilized DPP-IV for identification of antidiabetic ligands in lingonberry extract

**DOI:** 10.1371/journal.pone.0247329

**Published:** 2021-02-22

**Authors:** Rita de Cássia Lemos Lima, Ulrike Böcker, Gordon J. McDougall, J. William Allwood, Nils Kristian Afseth, Sileshi Gizachew Wubshet

**Affiliations:** 1 NOFIMA AS, Ås, Norway; 2 Plant Biochemistry and Food Quality Group, Environmental and Biochemical Sciences Department, The James Hutton Institute, Invergowrie, Dundee, Scotland, United Kingdom; Institute for Biological Research, SERBIA

## Abstract

In this work, a new magnetic ligand fishing probe for discovery of DPP-IV inhibitory ligands was developed and it was tested as a proof of concept on the fruit extract of *Vaccinium vitis-idaea* (lingonberry). The ligands were shown to have appreciable dipeptidyl peptidase IV (DPP-IV) inhibitory activity (IC_50_: 31.8 μg mL-1).) Inhibition of DPP-IV is a well-known therapeutic approach for management of type 2 diabetes (T2D). DPP-IV was successfully immobilized onto magnetic beads and was shown to retain its catalytic activity and selectivity over a model mixture. A total of four ligands were successfully fished out and identified as cyanidin-3-galactoside (***2***), cyanidin-3-arabinoside (***3***), proanthocynidin A (***4***), and 10-carboxyl-pyranopeonidin 3-*O*-(6″-*O*-p-coumaroyl)-glucoside (***5***) using HPLC/HRMS.

## 1. Introduction

Type 2 diabetes (T2D) is a metabolic disorder characterized by impaired function or production of insulin that manifests as a disturbance of glucose homeostasis. In healthy individuals, the increase of blood glucose levels after food intake leads to secretion of the incretin hormones glucagon-like peptide 1 (GLP-1) and glucose-dependent insulinotropic polypeptide (GIP). Once the incretin hormones bind to their receptors, a series of reactions (such as the potentiation of glucose-induced synthesis and secretion of insulin) take place in order to maintain the sugar homeostasis [1, 2]. However, GLP-1 and GIP are rapidly hydrolyzed by the action of the enzyme DPP-IV, a prolyl peptidase that exists in blood and is ubiquitously expressed on the apical surface of endothelial and epithelial cells. This will consequently constrain the effect of incretins on reducing blood sugar levels [2, 3]. Hence, inhibiting ligands of the alkaline protease DPP-IV are well-recognized as therapeutic agents that can be used for management of hyperglycemia in patients with T2D [4].

Berry fruits are widely considered as “superfoods”—low energy density foods, rich in vitamins, fibers and health promoting compounds–commonly used in traditional medicines to treat and prevent a number of diseases e.g. diabetes [4–6]. *Vaccinium vitis-idaea*, also known as lingonberry, is a wild berry belonging to the family Ericaceae used in traditional medicine in Northern Europe and in Cree communities in Canada to treat complications of T2D [7, 8]. The extract of lingonberry has been shown to have a hypoglycemic and anti-diabetic effect both *in vitro* and *in vivo*. These activities have been related especially to polyphenols, i.e. proanthocyanidins [9]; however, there are no reports assigning the antidiabetic activity of lingonberry to individual compounds in the extracts. Moreover, the mechanisms behind the antidiabetic effects of lingonberry have yet to be elucidated. Natural products are invaluable sources for pharmacologically active health promoting metabolites. In addition to inspiring the large majority of clinically approved drugs, the preparation of natural products has become increasingly attractive in the global market of functional foods and dietary supplements [10]. Despite their proven potential, preparations based on natural products are overly complex and documentation of pharmacological effects for constituents is hampered by a lack of suitable bioanalytical tools. The majority of *in vitro* screening techniques used in natural product-based drug discovery have relied on indirect detections involving reagents suitable for the available detectors (e.g., chromogenic or fluorogenic detections) [11]. This approach is particularly challenging when a complex mixture of natural products is to be screened. For instance, interferences due to the optical densities of samples and false positives due to nonspecific activity are among the prominent challenges in classical micro-plate based *in vitro* bioassays. Moreover, such assays must typically be accompanied by laborious bioassay-guided fractionation before the active principles can be identified [12].

Therapeutic targets immobilized on solid supports, such as superparamagnetic particles, have emerged as advanced bioanalytical tools for reliable and facilitated identification of bioactive molecules in complex mixtures. Affinity-based techniques have been successfully employed to investigate a wide range of biological systems contributing to the understanding of interactions like, antigen-antibody, receptor-ligand, enzyme-inhibitor/activator, and protein-protein [13]. Ligand fishing, one of the applications based on immobilized targets, is a technique used to selectively separate ligands (binders) of the immobilized therapeutic target from a complex mixture using magnetic force [14, 15]. In discovery of bioactive metabolites from natural products, ligand fishing has recently been recognized as a technique with significant promise [13]. Previous studies have demonstrated the use of immobilized therapeutic targets such as α-glucosidase [16] and α-amylase [12] on superparamagnetic beads and identification of antidiabetic compounds from *Eugenia catharinae* and *Ginkgo biloba*, respectively. Moreover, a recent study by Wubshet *et al*. has demonstrated that ligand fishing can also be used to rule out false positives from classical microplate-based assays [17]. Glutaraldehyde-based immobilization of therapeutic targets for ligand fishing application has mostly been performed under acidic conditions [12, 16–19]. Since DPP-IV is an alkaline protease, optimal activity of the enzyme cannot be retained in acidic conditions. However, immobilization of vital therapeutic targets such as the alkaline protease dipeptidyl peptidase IV (DPP-IV) has not been explored.

This study introduces a newly developed immobilized DPP-IV superparamagnetic beads for ligand fishing used for the identification of DPP-IV inhibitors in lingonberry extract for the first time. Through this study we also highlight the potential of lingonberry as source of antidiabetic constituents.

## 2. Materials and methods

### 2.1. Chemicals

DPP-IV from porcine kidney (EC 3.4.14.5) was purchased from Merck (Merck, Darmstadt, Germany). DPP-IV human recombinant (EC 3.4.14.5), Gly-pro-*p*-nitroanilide (GPPN), Tris, Diprotin A, HPLC-grade acetonitrile, formic acid, glutaraldehyde, hydroxylamine hydrochloride, sodium cyanoborohydride, ammonium acetate, HPLC-grade methanol, pyridine (99.8%), sitagliptin chloride, hippuric acid and ferulic acid were purchased from Sigma-Aldrich (St. Louis, MO, USA). Amine-terminated magnetic beads (30 mg/mL, 1μm) were purchased from Bioclone (San Diego, CA, USA). Water was purified by deionization and 0.22 μm membrane filtration using a Millipore system (Billerica, MA, USA), and other buffers were all analytical grades.

### 2.2. LB extract preparation and DPP-IV inhibition assay

An extract of lingonberry (*Vaccinium vitis-idaea* L.) was prepared essentially as reported previously [20]. Briefly, frozen wild harvested lingonberries obtained from Polarica AB (Haparanda, Sweden) in 2015 were extracted using 50% (v/v) aqueous acetonitrile containing 0.2% formic acid. The extract was reduced in volume by rotary evaporation to remove the acetonitrile then subjected to solid phase extraction on C18 units as described previously [20]. The SPE bound material was enriched in phenolics as measured by the Folin method and aliquots were dried in a speed-vac and frozen until further analysis.

The lingonberry (LB) extract was initially assayed in two concentrations: 50 μg mL^-1^ and 200 μg mL^-1^. The DPP-IV inhibition assay was performed according to a previous protocol [21] with slight modifications. Briefly, experiments were performed in triplicate in 96-well microplates to a final volume of 200 μL. To each well, 40 μL test sample, 45 μL of 50 mM Tris-HCl buffer (pH 7.5), and 15 μL of DPP-IV enzyme solution in Tris-HCl buffer pH 7.5 (0.05 U mL^-1^ final concentration) were added. The plate was incubated for 10 min at 37°C, and 100 μL of GPPN (0.2 mM in Tris-HCl, pH 7.5) was added to each well containing the mixture. The absorbance was subsequently measured at 405 nm every 1 min for 30 min, using a Synergy H1 hybrid multi-mode microplate reader (Biotek, Winooski, VT, USA). Diprotin A was used as a positive control. The percentage of DPP-IV inhibition was calculated using the following formula:

$$\%\;Inhibition=1-\left(\frac{{Slope}_{sample}}{{Slope}_{control}}\right)\times 100$$ (1)

where *Slope* is the Hill slope of the sample and of the negative control.

Finally, the DPP-IV IC_50_ value of lingonberry was determined using a dilution series of the extract (3.1 μg mL^-1^; 6.2 μg mL^-1^; 12.5 μg mL^-1^; 25.0 μg mL^-1^; 50.0 μg mL^-1^; 100.0 μg mL^-1^; 200.0 μg mL^-1^) and following the standard methods described above. The percentage of inhibition of DPP-IV was calculated as mean ± standard deviation using the above-described formula. The results were thereafter exported and used to assess the dose-response curves and IC_50_ values in GraphPad Prism, version 8.04 software (La Jolla, CA, USA). Data were fitted into the equation:

$$f\left(x\right)=min+\frac{max-min}{{1+(\frac{x}{{IC}_{50}})}^{slope}}$$ (2)

where min is the background, max-min is the y-range, x is the concentration and slope is the Hill slope.

### 2.3. Immobilization and characterization of DPP-IV

Initially, 5 mg of amino-terminated magnetic beads were washed (3 × 1 mL) with a coupling buffer consisting of a 10 mM phosphate buffer (pH 7.5, 25°C). Thereafter, 1 mL of a 5% glutaraldehyde solution was added to the Eppendorf containing the magnetic beads, and the mixture was rotated overnight at room temperature. After that, the beads were washed with coupling buffer (3 × 1 mL) and 1 mL of a solution of DPP-IV (human recombinant) at 4.0 U mL^-1^ was added to the magnetic beads and rotated for 48 hours in cold room (at 4°C). After magnetic separation, the supernatant was discarded and 1 mL of an endcapping solution (100 mM hydroxylamine, 2.5% NaBH_3_CN) was added to the magnetic beads. The mixture was kept under rotation for 24h at 4°C. Thereafter, the supernatant was discarded, and the DPP-IV linked magnetic beads (BcMag-DPP-IV) were washed (3 × 1 mL) with assay buffer (50 mM Tris-HCl buffer pH 7.5) and stored in the fridge in the same buffer.

#### 2.3.1. Fourier transform infrared (FTIR) microscopy

The *N*-terminus DPP-IV immobilized magnetic beads (MB-*N*-(DPP-IV)) were characterized using FTIR microscopy. FTIR spectra of MB-*N*-(DPP-IV) and a control with amine terminated magnetic beads (MB-NH_2_) were analyzed using a Perkin Elmer Spotlight 400 FTIR microscopy system. Thin films of magnetic bead in water suspension were deposited on IR-transparent CaF_2_-slides and briefly dried onto the slides. Spectra of the thin films were acquired using 32 scans and a resolution of 4 cm^−1^ in the spectral range from 4000 to 750 cm^−1^. Before the measurement of each sample spectrum, a background of the CaF_2_-slide was collected to account for water vapor and CO_2_. From each sample, a total of three spectra from different locations on the sample were acquired and averaged.

#### 2.3.2. Immobilized DPP-IV activity studies

Initially, 100 μL of Tris-HCl buffer and 100 μL of 0.4 mM of GPPN solution was added to a 1.5 mL micro-centrifuge tube containing 0.2 mg of MB-*N*-(DPP-IV) in 50 μL of assay buffer, and the mixture was incubated at 37° C for 30 minutes. In parallel, a similar experiment was performed with MB-NH_2_, as a negative control. After incubation, the supernatant was magnetically separated and transferred to a 96-well plate and the absorbance read at 405 nm. The experiment was performed on freshly prepared beads (day 0) and additional four activity measurements were performed using the same bead after storage (4°C) for 3, 5, and 9 days. All measurements were performed in triplicates. Normalized enzyme activities were calculated as a ratio of absorbance of the reaction product using MB-*N*-(DPP-IV) to absorbance of the reaction product using MB-NH_2_.

#### 2.3.3. DPP-IV enzyme equivalent activity

A dilution series of six concentrations of DPP-IV (from 0.12 U mL^-1^ to 0.003 U mL^-1^) in native state (non-bound) was prepared and 25 μL of each solution was transferred in triplicates to an Eppendorf tube together with 75 μL of 50 mM Tris-HCl buffer (pH 7.5) and 100 μL of a 0.2 mM solution of GPPN. The mixture was left to react for 30 minutes at 37°C under gentle shaking. Next, the solutions were transferred to a 96-well microplate; the absorbance of the samples was read at 405 nm and a calibration curve of the absorbances of the reaction product using DPP-IV in its native state was obtained. In parallel, 25 μL of a solution containing 0.05 mg of DPP-IV linked magnetic beads (theoretically equivalent to 10 mU of DPP-IV) was transferred to an Eppendorf tube in triplicate together with 75 μL of 50 mM Tris-HCl buffer (pH 7.5) and 100 μL of a 0.2 mM solution of GPPN. As mentioned above for non-bound DPP-IV, the mixture was left to react for 30 minutes at 37°C under gentle shaking. After the reaction time, the beads were magnetically separated, and the solution was transferred to a 96-well microplate and absorbance was read at 405 nm. The absorbance readings of the sample were interpolated in the calibration curve in order to calculate the equivalent activity of the immobilized DPP-IV in relation to DPP-IV in native state.

### 2.4. Ligand fishing using a model mixture

A model mixture of 5 μM of sitagliptin, diprotin A, hippuric acid and ferulic acid (equimolar) was prepared in buffer 50 mM Tris-HCl, pH 7.5 (S_0_) and used for the ligand fishing experiment. A volume of 600 μL of the model mixture was added to 1 mg of MB-*N*-(DPP-IV) and left to incubate for 10 minutes. After magnetic separation, the supernatant (S_1_) was saved and the magnetic beads were washed with 4 × 400 μL of assay buffer and wash solutions were saved after magnetic separation (S_2_-S_5_). Finally, the magnetic beads were eluted with 3 × 400 μL of 80% methanol in assay buffer (S_6_-S_8_).

The solutions S_0_-S_8_ were analyzed by LC-MS using an Agilent 1200 HPLC system comprising of a degasser, a quaternary pump, thermostatted autosampler, a column oven, a diode array detector. The samples were stored in the autosampler at 5°C and an injection volume of 10 μL was used. The column (Phenomenex Luna Omega Polar C18 100Å, 250 mm x 4.6 mm, 5 μm particle size; Phenomenex Ltd., USA) was operated at 25 °C and separation was performed using a flow rate of 0.5 ml min^-1^. The mobile phase consisted of H_2_O (solvent A) and acetonitrile (solvent B), both acidified with 0.1% formic acid. Separations were performed using the following gradient elution profile: 0 min, 0% B; 10 min, 50% B; 22 min, 100% B; 32 min, 100% B; 33 min, 0% B. The column eluate was directed to an Agilent 1100 LC/MSD Trap XCT Mass spectrometer equipped with an electrospray ionization (ESI) interface. Mass spectra were acquired both in positive and negative mode, using a drying temperature of 350 °C, a nebulizer pressure of 2 bar, a drying gas flow of 10 mL min^-1^, and a scan range from *m/z* 100 to 1000.

### 2.5. Ligand fishing of extract of lingonberry

A solution of 300 μg mL^-1^ of the LB extract was prepared in buffer 50 mM Tris-HCl, pH 7.5 (S_0_) and used for the ligand fishing experiment, in a similar fashion to the model mixture previously described. This LB solution (600 μL) was added to 1 mg of MB-*N*-(DPP-IV) and left to react for 10 minutes. After magnetic separation, the supernatant (S_1_) was saved and the magnetic beads were successively washed with 3 × 400 μL of assay buffer (S_2_-S_4_). Finally, the magnetic beads were eluted with 4 × 400 μL of 80% methanol in assay buffer (S_6_-S_8_). The supernatants S_0_-S_8_ were analyzed using the same LC-MS instrument as described previously (section 2.4). All experimental parameters, with the exception to the elution gradient, were similar. The following elution gradient was used for analysis of S_0_-S_8_ from ligand fishing of LB extract: 0 min, 5% B; 10 min, 25% B; 15 min, 27% B; 30 min, 37% B; 32 min, 50% B; 35 min, 100% B; 40 min, 100%B; 42 min, 5% B.

### 2.6. Identification of DPP-IV ligands from lingonberry using HPLC-HRMS

The lingonberry extract was analyzed using an LC-Q-TOF system consisting of a 1260 HPLC equipped with a photodiode-array detector (DAD), coupled to a 6510 Q-TOF mass spectrometer with ESI ion source and controlled by MassHunter software version B.07.00 (all Agilent, Santa Clara, CA, USA). The column, injection volume, flow rate, mobile phase composition, temperature and gradient elution profile were as described in section 2.5. MS spectra in the range *m/z* = 60 to m/z = 1000 were acquired in both negative and positive ion mode, using a drying temperature of 365°C, a nebulizer pressure of 2 bar, and a drying gas flow of 13 L min^-1^. Mass data were automatically corrected internally against a reference mass solution. MS/MS spectra in the range *m/z* = 20 to *m/z* = 1500 were acquired with the same chromatographic and spectrometric settings, with fragmentation energies set to consensus 10 V, 20 V and 40 V. The five peaks pinpointed as the fished-out binding ligands of DPP-IV were targeted and analyzed based on retention time and *m/z*.

## 3. Results and discussion

### 3.1. Synthesis and characterization of immobilized DPP-IV magnetic beads

**Fig 1:**
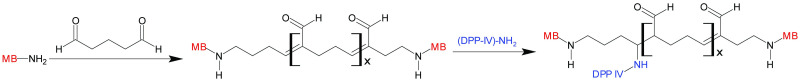
Proposed immobilization mechanism of DPP-IV on magnetic beads (MB) at pH = 7.4.

The immobilization of DPP-IV was performed by linking the *N*-terminus end of the enzyme to the aldehyde-terminated short-arm magnetic beads (BcMag). Following that, a Borch reduction was carried out to endcap and stabilize the unbound aldehyde terminals of the linker glutaraldehyde. Glutaraldehyde-based immobilization of therapeutic targets for ligand fishing application has mostly been performed under acidic conditions [12, 16–19]. However, since DPP-IV is an alkaline protease, optimal activity of the enzyme could not be retained in acidic conditions. Therefore, an immobilization process was developed using 10 mM phosphate (pH 7.5, 25°C) as coupling buffer. Unlike acidic conditions, under slightly basic conditions, as used in the current study, intramolecular aldolic condensations are expected to result in a polymeric form of glutaraldehyde on the surface of the beads, as shown in Fig 1. Immobilization may then occur through formation of Schiff bases between internal aldehyde groups of the polymeric form of glutaraldehyde and primary amino groups of DPP-IV (Fig 1) [22].

**Fig 2:**
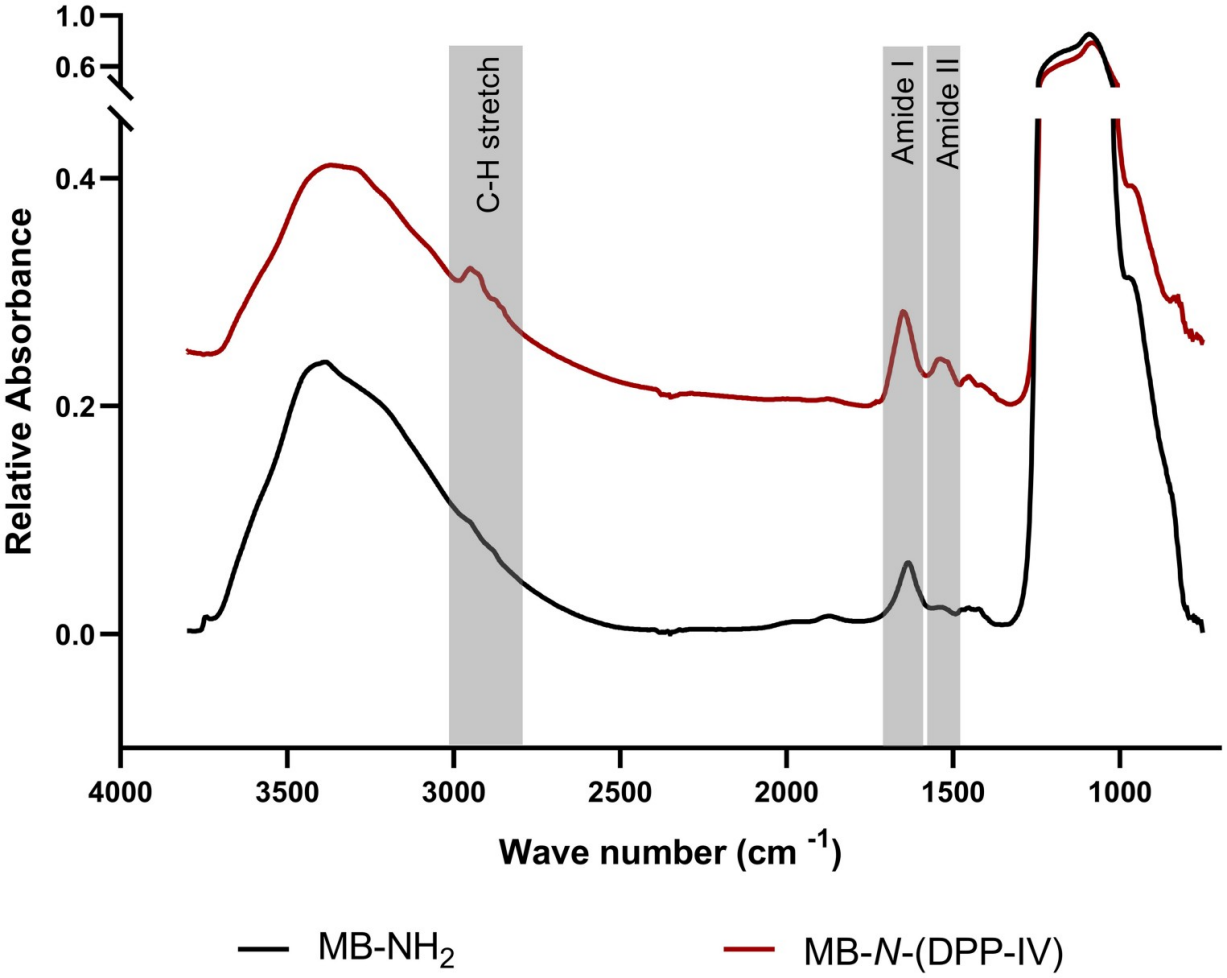
FTIR spectra of a control amine terminated magnetic beads (MB-NH_2_) and *N*-terminus DPP-IV immobilized magnetic beads (MB-*N*-(DPP-IV)) acquired using FTIR microscopy.

The *N*-terminus DPP-IV immobilized magnetic beads (MB-*N*-(DPP-IV)) were characterized using FTIR microscopy. Average FTIR spectra of MB-*N*-(DPP-IV) and of a control with amine terminated magnetic beads (MB-NH_2_) are shown in Fig 2. For both the samples the most dominating feature in the spectrum is the region from 900–1300 cm^-1^, which is due to asymmetric (~1180 cm^-1^) and symmetric (~1080 cm^-1^) stretching vibrations of the Si-O-Si siloxane rings and the Si-O(H) stretching at 960 cm^-1^ [23]. Yet, compared to the FTIR spectrum of MB-NH_2_, that of MB-*N*-(DPP-IV) very clearly reveals the amide I and II bands characteristic for proteins found at ~1648 cm^-1^ (amide-I) and ~1530 cm^-1^ (amide-II), while the spectrum of MB-NH_2_ only displays a band at 1630 cm^-1^ likely caused by the -OH bending of adsorbed water [17]. This shows that the immobilized enzyme has been successfully attached to the magnetic beads. Moreover, distinct vibrational bands from polymeric aliphatic structures were observed at 2800–3000 cm^-1^, i.e. the CH-stretch region. This is consistent with the proposed polymeric form of glutaraldehyde appearing on the magnetic beads as suggested above.

### 3.2. Activity of the immobilized DPP-IV magnetic beads

**Fig 3:**
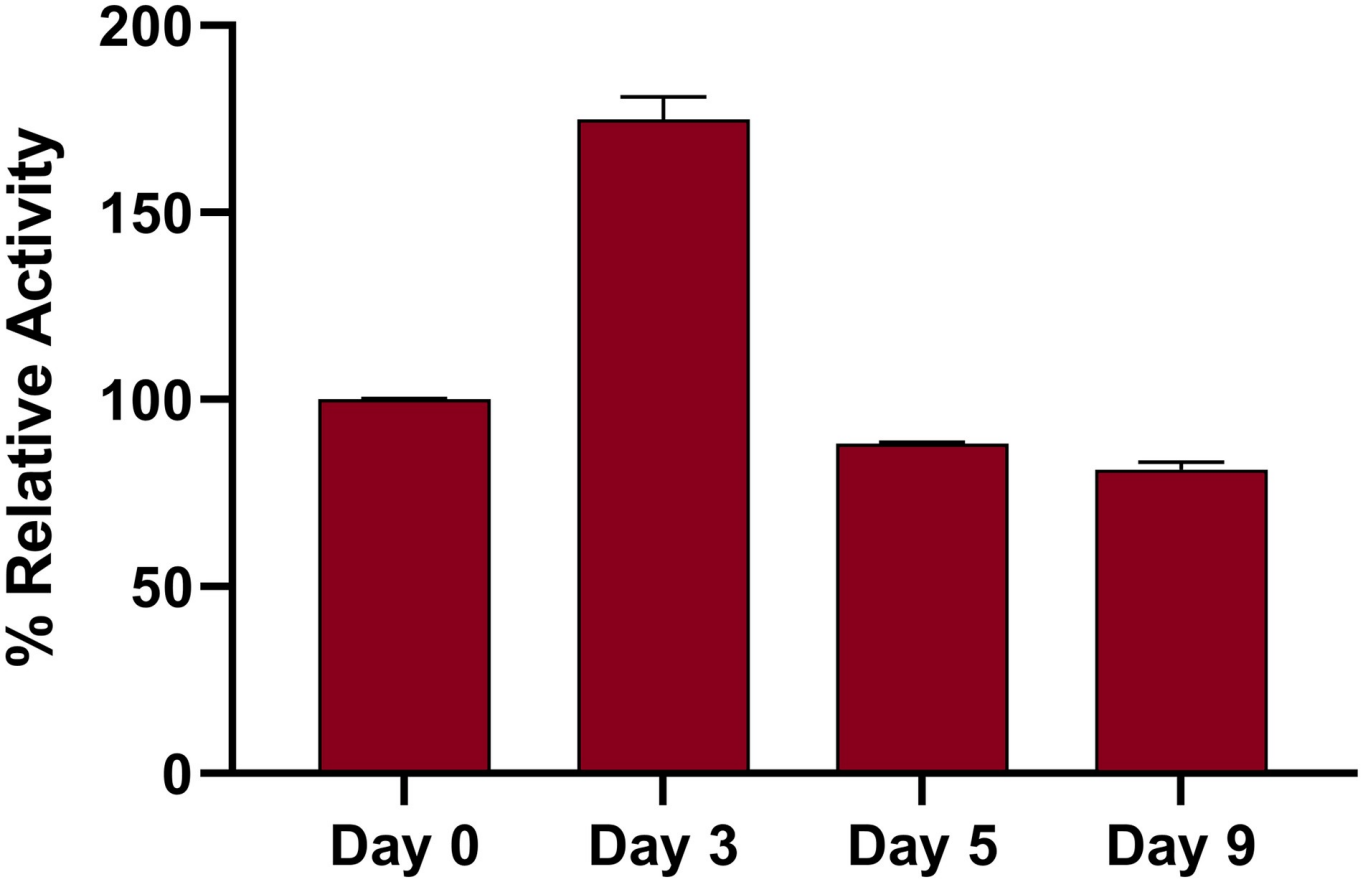
Observed catalytic activity of MB-*N*-(DPP-IV) over a period of nine days. Activity of the freshly prepared enzyme was set to 100% for the sake of normalized comparison. Experiments were performed in triplicates using the same batch of immobilized enzymes that were kept at 4°C. Bars represent the standard deviation.

The catalytic activity of the immobilized DPP-IV magnetic beads was evaluated using the amine functionalized magnetic beads (i.e., MB-NH_2_) as a control. The assay was performed using the standard substrate Gly-Pro-*p*-nitroanilide (GPPN) and activity was measured by monitoring production of nitroanilide. The results confirmed that the MB-*N*-(DPP-IV) beads retained activity and the immobilization of the catalytically active form of DPP-IV (Fig 3). To further investigate potential reusability of the immobilized DPP-IV, activity was measured four times within nine days. The results show that after nine days of storage and four cycles of activity measurement, MB-*N*-(DPP-IV) only lost 18.8% of its initial activity. Interestingly, the activity appeared to increase at day 3 compared to freshly prepared MB-*N*-(DPP-IV). While it is common that immobilization increases stability and activity of enzymes the observed increase after three days of storage was peculiar. Overall, these results show that the MB-*N*-(DPP-IV) are stable at 4°C and can maintain catalytic activity through multiple cycles of re-use.

The relative catalytic activity of MB-*N*-(DPP-IV) was further evaluated and compared with the free form of (i.e., not immobilized) DPP-IV. The relative activity of 0.01 U of immobilized DPP-IV was found to be equivalent to that of 0.083 U mL^-1^ native DPP-IV, which indicated a > 8-fold increase in activity when the DPP-IV is in an immobilized form. Previous studies have also shown that immobilization of enzymes on solid supports can improve stability and consequently catalytic activity [12, 24]. Overall, these results suggest that the immobilization protocol established in the present study did not negatively affect the activity of DPP-IV.

### 3.3. Ligand fishing from a test model mixture

To evaluate the performance of the MB-*N*-(DPP-IV) beads for a ligand fishing application, a mixture containing both known ligands and non-ligands of DPP-IV was prepared and used as a model mixture. Sitagliptin, a DPP-IV inhibitor drug in clinical use [25] and diprotin A, a well-known DPP-IV inhibitor [26], were chosen as binders, whereas hippuric acid and ferulic acid were selected as non-binders.

**Fig 4:**
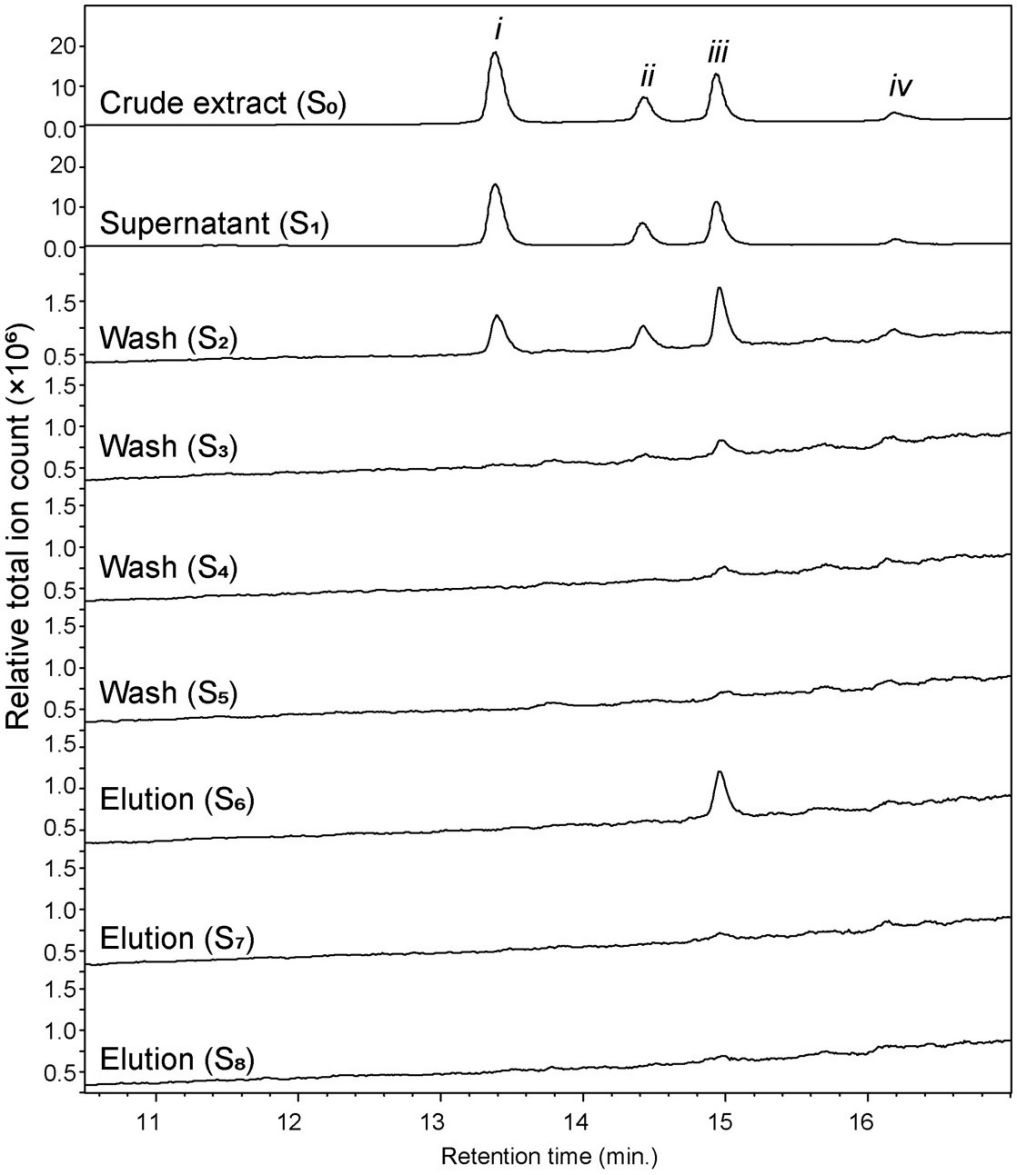
Overlaid total ion chromatograms acquired for different solutions (S_0_−S_8_) obtained from MB-*N*-(DPP-IV)-based magnetic ligand fishing of a model mixture consisting of diprotin A (*i*), hippuric acid (*ii*), sitagliptin (*iii*), and ferulic acid (*iv*). Sitagliptin(***iii***) was identified as high affinity ligands of DPP-IV.

The LC-MS chromatograms (section 2.4) of the model mixture (S_0_), the supernatant after incubation (S_1_), four consecutive washings (S_2_-S_5_) and three consecutive elutions (S_6_-S_8_) are presented in Fig 4. The chromatogram from S_0_ shows four distinct peaks, as expected, with *m/z* 340.4 at RT 13.2 min (*i*, *m/z* [M-H]^-^ for diprotin A), 177.7 at RT 14.8 min (*ii*, *m/z* [M-H]^-^ for hippuric acid), 442.2 at RT 15.0 min (*iii*, *m/z* [M-H]^-^ for sitagliptin), and 194.3 at RT 16.3 min (*iv*, *m/z* [M-H]^-^ for ferulic acid). All four peaks were also detected in the supernatant (S_1_) and in the first wash (S_2_). This is typical, as both ligands and non-binders were added in excess of the available interaction sites. In the following wash cycles (S_3_-S_5_), intensities of all peaks were gradually decreasing. Interestingly, in the first methanol elution (S_6_) only sitagliptin (peak *iii*) could be confirmed, with adequate peak intensity, as a fished-out ligand. This is despite the fact that the test mixture contained another binding ligand (i.e., diprotin A). This could be due to the fact that sitagliptin (K_i_: 8.9 nM; IC_50_: 18.0 nM) is a more potent ligand than diprotin A (K_i_: 2.2 μM; IC_50_: 24.7 μM) [27–30]. Hence, at the equimolar concentrations used in the current study and with three orders of magnitude higher affinity of sitagliptin compared to diprotin A, sitagliptin will expectedly saturate the available binding sites. This finding shows that MB-*N*-(DPP-IV) are both efficient in fishing ligands, but also are selective towards the most potent binding ligands in a mixture.

### 3.4. Ligand fishing of lingonberry extract

The DPP-IV inhibition potential of a lingonberry extract was assessed before it was used in a subsequent ligand fishing experiment. The LB extract showed an IC_50_ value of 31.8 μg mL^-1^ for DPP-IV inhibition (S2 Fig). Although lingonberry extracts have been previously reported with antidiabetic activities [7–9], DPP-IV inhibition has not been previously noted for lingonberry, but DPP-IV inhibition has been noted in extracts of other berry species [31]. Thus, this is the first report of a lingonberry extract with DPP-IV inhibitory effect, moreover, this is the first report of the identification of individual antidiabetic compounds targeting DPP-IV from this plant species.

**Fig 5:**
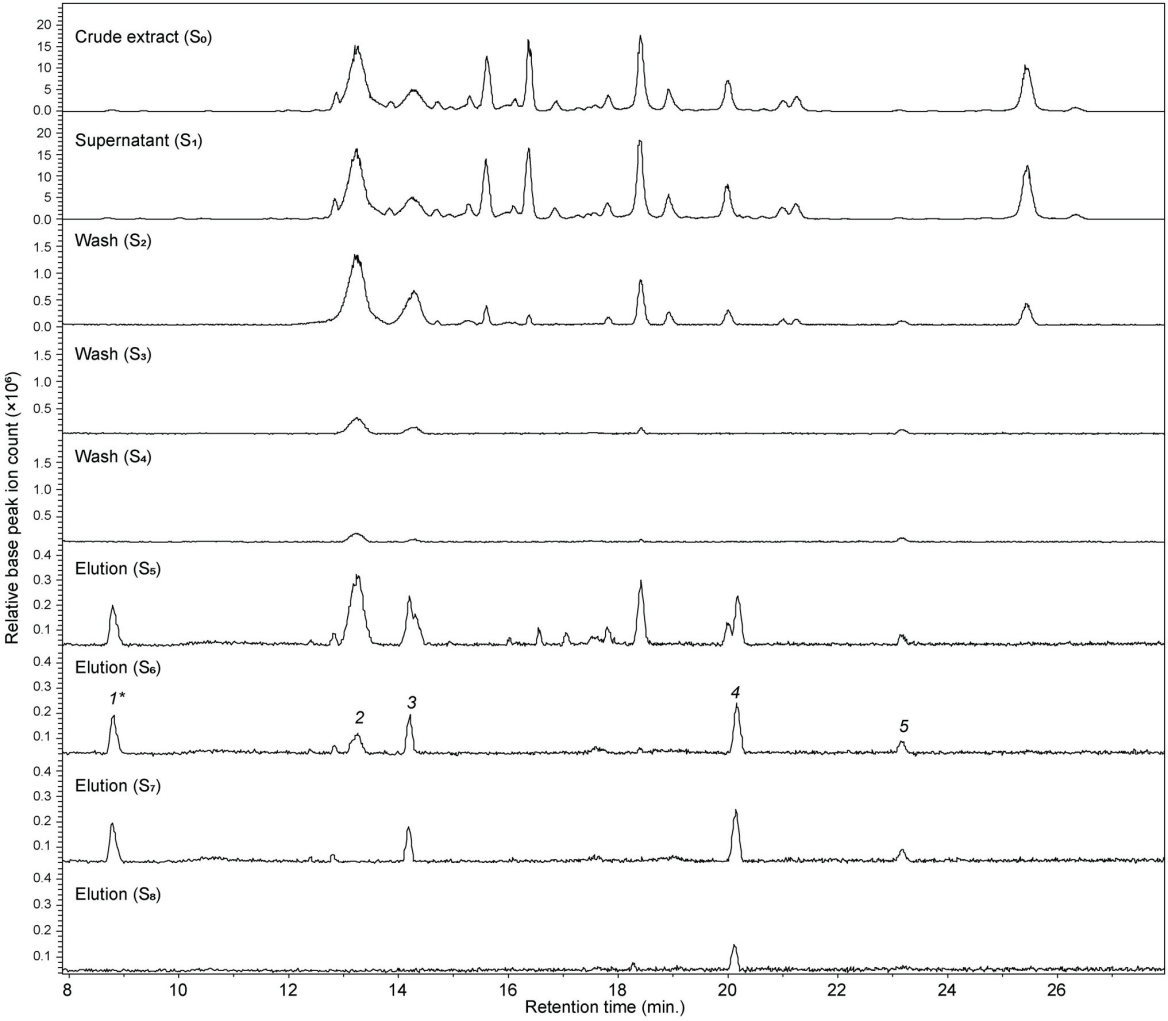
Overlaid base peak chromatograms acquired for different solutions (S_0_−S_8_) obtained from MB-*N*-(DPP-IV)-based magnetic ligand fishing of lingonberry extract. Peaks **2, 3, 4,** and **5** were identified as high affinity ligands of DPP-IV.

A second ligand fishing experiment was carried out as a proof of concept with the lingonberry extract to identify candidate DPP-IV ligands. The base peak chromatograms from LC-MS analysis (section 2.5) of the crude extract loaded on the magnetic beads (S_0_), the supernatant after ligand fishing (S_1_), the washings (S_2_-S_4_) and the elutions (S_5_-S_8_) are shown in Fig 5. The crude extract (S_0_) chromatogram showed the presence of seven major peaks and several minor peaks. The overall profile was similar to previous reports [32]. The supernatant (S1) chromatogram of the solution incubated for 10 minutes with MB-*N*-(DPP-IV) presented a roughly similar profile as S_0_, which was expected as there will be an excess amount of all analytes for the available binding sites of the immobilized DPP-IV.

In the subsequent washing steps S_2_ and S_3_, the majority of the compounds observed in S_1_ were washed off as was evident from the chromatogram of the washing step S_4_, which only showed three minor peaks. However, many of the peaks from the original mixture were observed again in the chromatogram acquired for the first methanol elution (S_5_). This is most likely a result of other non-specific interactions, such as hydrophobic interaction, of analyte molecules with the magnetic bead which have retained many of the original constituents present. One issue when performing ligand fishing experiments is the difficulty of finding a “‘true” negative control that can exclude by comparison false positives or non-specific ligands. In order to avoid a mislead of results, in the present study, the chromatogram from the second methanol elution S_6_ was considered for the identification of ligands as S_6_ is more likely to pinpoint the potential DPP-IV binding ligands from the lingonberry extracts. The chromatogram from S_6_ contained five distinct chromatographic peaks (**1–5**) which were later targeted and analyzed using LC-HRMS as potent DPP-IV ligands. Interestingly, only four peaks (i.e. **1, 3, 4** and **5**) of the five targeted peaks were retained until S_7_ and peak 4 was further observed in last elution step (S_8_). This suggests that the compound eluted in peak 4 has potentially high affinity towards DPP-IV.

Targeted mass spectrometry analysis of the five peaks (**1–5**) was performed directly from the original LB extract. The compound eluted in peak 1 could not be detected in the original extract and hence was ruled out as a potential contaminant from the fishing procedure. After studying the MS data (both molecular ions in positive and negative mode and their diagnostic fragment ions) and comparing with literature, the latter four ligands were identified as cyanidin-3-galactoside (peak 2), cyanidin-3-arabinoside (peak 3), proanthocyanidin A (peak 4) and 10-carboxyl-pyranopeonidin 3-*O*-(6″-*O*-p-coumaroyl)-glucoside (peak 5) [Table 1].

**Table 1:** LC-MS/MS data of the identified DPP-IV ligands from lingonberry extract.

Peak	RT (min)	*m/z* [M + H]^+^ (MF, ppm)	MS/MS (*m/z*)	*m/z* [M - H]^-^ (MF, ppm)	MS/MS (*m/z*)	Compound	Reference
2	13.3	449.1093 [M]^+^ (C_21_H_21_O_11_^+^, Δ -1.26 ppm)	287.0760	447.0883 [M]^-^ (C_21_H_19_O_11_^-^, Δ -1.98 ppm)	112.9856	Cyanidin-3- galactoside	[33]
3	14.2	419.1077 [M]^+^ (C_20_H_19_O_10_^+^, Δ -1.26 ppm)	287.0532	417.0773 [M]^-^ (C_20_H_17_O_10_^-^, Δ -1.39 ppm)	255.0697; 112.9856	Cyanidin-3-arabinoside	[33]
4	20.2	577.1517 [M+H]^+^ (C_30_H_25_O_12_^+^, Δ -1.82 ppm)	-	575.1190 [M -H]^-^ (C_30_H_23_O_12_^-^, Δ 0.87 ppm)	539.0819; 407.0671; 289; 285.0354	Proanthocyanidin A	[33]
5	23.2	677.1506 [M+H]^+^ (C_34_H_29_O15^+^, Δ -0.74 ppm)	367.6630; 359.4109; 120.9426	675.0 [M - H]^-^ (C_34_H_27_O_15_^-^, Δ 0.81 ppm)	713.5 [M + Cl]^-^; 723.4 [M + COOH]^-^; 112.9	10-carboxyl-pyranopeonidin 3-*O*-(6″-O-p-coumaroyl)-glucoside	[34]

Retention time, MS and MS/MS data are presented with the references of compounds.

The anthocyanins cyanidin-3-galactoside, cyanidin-3-arabinoside and proanthocyanidin A have previously been identified from extracts of *Vaccinium vitis-idaea* [35]. The compound which eluted as peak 5 (10-carboxyl-pyranopeonidin 3-*O*-(6″-*O*-p-coumaroyl)-glucoside) has been identified as *α*-glucosidase inhibitor from grape pomace [34]. Cyanidin-3-galactoside has been reported as a moderate DPP-IV inhibitor with an IC_50_ value of 0.42 μM, and it has also shown α-glucosidase inhibitory activity with an IC_50_ of 479.8 μM [31, 36]. Interestingly, the compound identified as the ligand with highest DPP-IV affinity in the present work (i.e. peak 4; proanthocyanidin A) has also been recognized as a potent DPP-IV inhibitor in a previous study by Johnson *et al*. [37]. In the study, a proanthocyanidin A enriched (PAC enriched) fraction from blueberry was shown to inhibit DPP-IV with an IC_50_ value of 16.6 μM. This demonstrates that compounds identified as high affinity ligands using the developed ligand fishing technology are in fact promising inhibitors of DPP-IV.

## 4. Conclusion

In the present study a new magnetic ligand fishing method was developed for rapid identification of DPP-IV inhibiting ligands from a lingonberry extract. An immobilization protocol for the alkaline protease DPP-IV was developed using glutaraldehyde as linker. After characterization of the immobilized therapeutic target, a proof-of-concept study was conducted using both a model mixture and the lingonberry extract. In both cases, DPP-IV inhibiting ligands were successfully retained and fished out. Interestingly, the successive elution procedure suggested that ligands with high affinity could be retained later in the elution steps. Although the present novel approach is suitable for identification of DPP-IV inhibitory ligands, some issues must still be addressed in future studies. This includes the difficulty of finding a “true” negative control, as the options available today can mislead the results as for non-specific binding. This presented an excellent opportunity to target high affinity ligands in plant extracts, such as the superfood lingonberry. Targeted LC-MS/MS analysis of the ligands from lingonberry led to identification of four metabolites, three of which have previously been reported as DPP-IV inhibitors. In conclusion, we have successfully identified DPP-IV inhibiting ligands from lingonberry extract using a new screening ligand fishing technology. This technology presents an excellent tool to screen potential antidiabetic metabolites in complex extracts of food matrices.

## Supporting information

S1 Fig Calibration curve obtained for a dilution series of DPP-IV enzyme in native state used to establish the equivalent activity of immobilized DPP-IV on magnetic beads. (DOCX)

S2 Fig IC_50_ curve obtained for DPP-IV inhibitory effect of the extract of lingonberry. The experiment used a dilution series of seven concentrations performed in triplicates. The bars represent the standard deviation of each datapoint.
(DOCX)
