# Supplementary material for: Magnetic ligand fishing using immobilized DPP-IV for identification of antidiabetic ligands in lingonberry extract
Source: PLoS One. 2021 Feb 22;16(2):e0247329. doi: 10.1371/journal.pone.0247329 (PMC7899330; doi:10.1371/journal.pone.0247329)
Supplement: S1 Fig — (DOCX) [file pone.0247329.s001.docx]

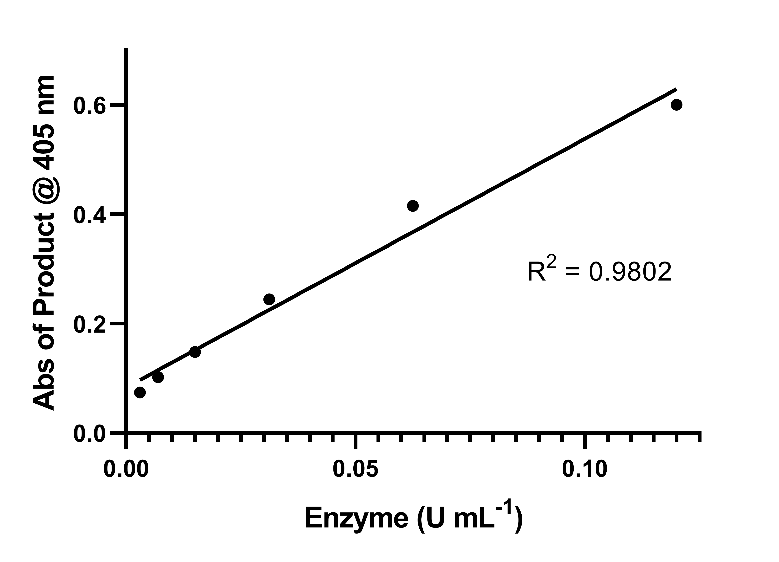


**S1 Fig.** Calibration curve obtained for a dilution series of DPP-IV enzyme in native state used to establish the equivalent activity of immobilized DPP-IV on magnetic beads.
