# Supplementary material for: Magnetic ligand fishing using immobilized DPP-IV for identification of antidiabetic ligands in lingonberry extract
Source: PLoS One. 2021 Feb 22;16(2):e0247329. doi: 10.1371/journal.pone.0247329 (PMC7899330; doi:10.1371/journal.pone.0247329)
Supplement: S2 Fig — The experiment used a dilution series of seven concentrations performed in triplicates. The bars represent the standard deviation of each datapoint. (DOCX) [file pone.0247329.s002.docx]

**S2 Fig.** IC_50_ curve obtained for DPP-IV inhibitory effect of the extract of lingonberry. The experiment used a dilution series of seven concentrations performed in triplicates. The bars represent the standard deviation of each datapoint.
